# D1R/GluN1 complexes in the striatum integrate dopamine and glutamate signalling to control synaptic plasticity and cocaine-induced responses

**DOI:** 10.1038/mp.2014.73

**Published:** 2014-07-29

**Authors:** E Cahill, V Pascoli, P Trifilieff, D Savoldi, V Kappès, C Lüscher, J Caboche, P Vanhoutte

**Affiliations:** 1 INSERM, UMR-S 1130, Neuroscience Paris Seine, Paris, France; 2 CNRS, UMR 8246, Neuroscience Paris Seine, Paris, France; 3 Sorbonne Universités, UPMC Université Paris 06, UMR-S 8246, Neuroscience Paris Seine, Paris, France; 4 Department of Basic Neurosciences, Medical Faculty, University of Geneva, Geneva, Switzerland; 5 Nutrition and Integrative Neurobiology, INRA UMR 1286, Bordeaux, France; 6 University of Bordeaux, Bordeaux, France; 7 Center for Neuroscience. Columbia University, Kolb Research Building, New York, NY, USA; 8 Department of Clinical Neuroscience, Geneva University Hospital, Geneva, Switzerland

## Abstract

Convergent dopamine and glutamate signalling onto the extracellular signal-regulated kinase (ERK) pathway in medium spiny neurons (MSNs) of the striatum controls psychostimulant-initiated adaptive processes underlying long-lasting behavioural changes. We hypothesised that the physical proximity of dopamine D1 (D1R) and glutamate NMDA (NMDAR) receptors, achieved through the formation of D1R/NMDAR complexes, may act as a molecular bridge that controls the synergistic action of dopamine and glutamate on striatal plasticity and behavioural responses to drugs of abuse. We found that concomitant stimulation of D1R and NMDAR drove complex formation between endogenous D1R and the GluN1 subunit of NMDAR. Conversely, preventing D1R/GluN1 association with a cell-permeable peptide (TAT-GluN1C1) left individual D1R and NMDAR-dependent signalling intact, but prevented D1R-mediated facilitation of NMDAR–calcium influx and subsequent ERK activation. Electrophysiological recordings in striatal slices from mice revealed that D1R/GluN1 complexes control the D1R-dependent enhancement of NMDAR currents and long-term potentiation in D1R-MSN. Finally, intra-striatal delivery of TAT-GluN1C1 did not affect acute responses to cocaine but reduced behavioural sensitization. Our findings uncover D1R/GluN1 complexes as a major substrate for the dopamine–glutamate interaction in MSN that is usurped by addictive drugs to elicit persistent behavioural alterations. They also identify D1R/GluN1 complexes as molecular targets with a therapeutic potential for the vast spectrum of psychiatric diseases associated with an imbalance between dopamine and glutamate transmission.

## Introduction

Addiction is a chronic and relapsing psychiatric disorder, thought to occur in vulnerable individuals due to a perturbation of goal-directed behaviour.^1^ The striatum orchestrates motivated behaviour such as motor planning and reward-dependent learning.^2,3^ These functions require the integration, by the medium spiny neurons (MSNs) of the striatum, of glutamate inputs arising from the cortex and thalamus together with dopamine (DA) release. It is widely acknowledged that DA and glutamate systems interact to control synaptic plasticity in MSN and addiction-related behaviour, but the underlying molecular mechanisms are still poorly understood. Herein, we hypothesised that, because of their physical proximity, DA and glutamate receptor complexes play the role of detectors of coincidence for DA and glutamate signals in the striatum thereby participating to persistent behavioural adaptations induced by drugs of abuse.

All addictive drugs increase DA in the nucleus accumbens (NAc) region of the striatum,^4^ where it modulates glutamate transmission.^5,6^ As such, drugs of abuse usurp the neural reward circuitry and induce molecular events underlying long-lasting changes in synaptic transmission^7^ and behaviour.^8^ Drugs of abuse also share the ability to activate extracellular signal-regulated kinase (ERK) in the striatum,^9^ where it controls gene expression, long-term synaptic plasticity and behaviour.^10^ Activation of ERK by cocaine occurs in dopamine D1 receptor (D1R)-expressing MSN^11^ (D1R-MSN) and behaves as an integrator of DA and glutamate signals as it requires the coincident stimulation of D1R and glutamate NMDA receptors^12,13^ (NMDAR). We recently established a pivotal role of the signalling crosstalk between D1R and NMDAR in cocaine-mediated responses, as D1R stimulation in absence of glutamate does not trigger ERK activation but can potentiate NMDAR-dependent calcium influx via the tyrosine phosphorylation of GluN2B subunits. This interplay between D1R and GluN2B-NMDAR was necessary for cocaine-induced ERK activation and long-term behavioural alteration.^14^ Indeed, a causal connection between ERK-dependent long-term potentiation (LTP) in the NAc and behavioural sensitization was recently demonstrated.^15^

A number of studies have demonstrated that the physical interaction between D1R and NMDAR subunits was dynamically regulated by ligands and can mutually modify receptor function,^16^ thereby increasing the signalling diversity and complexity. Co-immunoprecipitation, GST-pull down and resonance energy transfer techniques demonstrated that the D1R C-terminal tail binds to the GluN1 and GluN2A subunits.^17, 18, 19^ *In vivo*, uncoupling the D1R/NMDAR receptor complexes in the hippocampus impairs NMDA-dependent LTP and working memory.^20^ The striatum is the main target of DA projections in the brain and striatal-dependent behaviour greatly depends on DA and glutamate crosstalk, yet the functional role of the D1R/NMDAR interaction for striatal signalling, plasticity and responses to addictive drugs is unknown.

We optimised an interfering peptide-based strategy to selectively disrupt endogenous D1R/GluN1 complexes in neurons. We found that a TAT-coupled peptide (TAT-GluN1C1), corresponding to the C1 cassette of GluN1 that binds to the t2 domain of D1R^18,19^ blocked signalling pathways downstream of D1R/GluN1 complexes, yet preserved individual D1R and NMDAR signalling. TAT-GluN1C1 blocked D1R-dependent potentiation of calcium influx through GluN2B-NMDAR along with ERK activation in MSN after a co-stimulation (co-stim) of D1R and NMDAR, which favoured endogenous D1R/GluN1 heteromerization. As predicted by ERK inhibition, TAT-GluN1C1 prevented NMDAR-mediated LTP of glutamate transmission onto D1R-MSN. *In vivo,* TAT-GluN1C1 preserved basal locomotor activity and acute responses to cocaine, but impaired the development of behavioural sensitization. Our findings identify D1R/GluN1 complexes as a molecular bridge by which DA modulates glutamate transmission and glutamate-dependent plasticity and, eventually, behaviour in response to cocaine.

## Materials and methods

### Chemicals and reagents

(R)-(+)-SKF38393 (Sigma Aldrich, St Louis, MO, USA) and/or L-glutamic acid (Calbiochem, San Diego, CA, USA) were diluted at the indicated concentrations in purified water. When indicated, the following cell-penetrating peptides were administered 1 h before and during further treatments: TAT-GluN1C1: GRKKRRQRRRPPDRKSGRAEPDPKKKATFRAITSTLASSFKRRRSSKDT; its inactive counterpart, the TAT-GluN1C1Δ: GRKKRRQRRRPPDRKSGRAEPDPKKKATFRAITSTLASDT, TAT-D1-t2: GRKKRRQRRRPPLVYLIPHAVGSSEDLKREEAGGIPKPLEKL, TAT-D1-t3: GRKKRRQRRRPPSPALSVILDYDTDVSLEKIQPVTHSGQHST and TAT-GluN1C1min: GRKKRRQRRRPPSFKRRRSSK, (IBPS, Institut de Biologie Paris - Seine), UMPC, Paris; France). For the *in vitro* studies, peptides were administered at a final concentration of 5 μM 1 h prior and during pharmacological treatments. For *in vivo* experiments, the peptides were infused at a concentration of 5 pmol per hemisphere in a volume of 0.5 μl, 1h before the administration of cocaine. Other pharmacological agents used are detailed as Supplementary Information.

### Proximity ligation assay

Brain slices were prepared as previously described^21^ and proximity ligation assay (PLA) was performed using the Duolink *in situ* kit (Olink Bioscience, Uppsala, Sweden) according to the manufacturer's instructions with the following modifications: PLA probes incubation was for 2 h; ligation was performed for 45 min; amplification step was extended for 2 h with a concentration of polymerase of 1/60 all at 37°C. Striatal primary cultured cells were plated into 8-μ well plates (LabTek, Dutscher, Brumath, France). Blocking (1 h at room temperature) and primary antibody (overnight at 4 °C) incubations were performed in a 3% bovine serum albumin (Sigma Aldrich) and 0.2% Triton X-100 solution. Rabbit anti-GluN1 (ab17345, Abcam, Cambridge, MA, USA) and rat anti-D1R (D2944, Sigma Aldrich) were diluted (1:500) in the blocking solution. The anti-rabbit (+) PLA probe (1:5) along with an anti-rat (−) probe (1:100) were diluted in the blocking solution. Anti-rat PLA probes were generated according to the manufacturer's instructions using the Duolink Probemaker (Olink Bioscience). Goat-anti-rat IgGs (Santa Cruz, Heidelberg, Germany) were used. Cells were mounted in FluorSave (Calbiochem). Image acquisition and quantification are detailed in the Supplementary Information section.

### Electrophysiology

Heterozygous transgenic mice in which tdTomato expression was driven by D1R (*drd1a-tdTomato* from Jackson Laboratories, Sacramento, CA, USA) gene regulatory element or enhanced green fluorescent protein (eGFP) expression was driven either by D1R (*drd1a*-eGFP) or D2R (*drd2*-eGFP) gene regulatory elements^22^ were backcrossed in C57Bl/6 mice for 3–4 generations and housed in groups of 3–4 in a temperature- and hygrometry-controlled environment with a 12/12- h light/dark cycle. All procedures were approved by the Institutional Animal Care and Use Committee of the University of Geneva.

Slice preparation and high-frequency stimulation-induced AMPAR-LTP were performed as described.^15^ For NMDAR-mediated excitatory post synaptic currents (EPSCs), the internal solution contained (in mM): 130 cesium chloride, 4 NaCl, 5 sodium creatine-phosphate, 2 MgCl_2_, 2 Na2ATP, 0.6 Na3GTP, 1.1 EGTA, 5 HEPES, 0.1 spermine (see Supplementary Materials and Methods for information about pharmacological treatments).

For the LTP experiments, the internal solution contained (in mM): 140 k-gluconate, 5 KCl, 10 HEPES, 0.2 EGTA, 2 MgCl2, 4 Na2ATP, 0.3 Na3GTP and 10 sodium creatine-phosphate. NMDAR-EPSCs were isolated at −70 mV by omitting MgCl_2_ from the ACSF and adding the AMPAR antagonist. All experiments were carried out in the presence of picrotoxin (100 μM).

### Animals, drugs administration and locomotor sensitization

Male 7-week-old C57BL/6 mice (Janvier laboratories, Le Genest St Isle, France) were individually housed with *ad libitum* access to food and water and maintained on a 12/12 -h light/dark cycle and habituated to the animal facility for 1 week before experiments. Experiments took place during the animal's light cycle. Animal work was carried out in accordance with the standard ethical guidelines (European Community guidelines on the care and use of laboratory animals: 2010/63/EU).

Using isofluorane anaesthesia, 22 gauge stainless-steel guide cannulae (Plastics One, Roanoke, VA, USA) were implanted bilaterally (antero–posterior +1.5; medio–lateral ±1.6; dorso–ventral 4.1; 13° angle) and fixed with support screws and dental cement (Brudentaire, Paris, France). Mice recovered during 1 week with daily handling. One hour before intra-peritoneal cocaine injections (15 mg kg^−1^; Sigma Aldrich), 5 pmol of cell-penetrating peptide were bilaterally infused (0.5 μl; 0.2 μl min^-1^) into the NAc.

Locomotor activity (1/4 turns) was measured automatically as the number of crossings between quarters of a circular corridor using ANY-Maze software (ANY-Maze, Stoeling, Wood Dale, IL, USA) and locomotor sensitization was performed as previously described.^13^ Spontaneous locomotor activity was first measured (day 1), over 15 min and 1 h after a bilateral infusion of TAT-GluN1C1 or TAT-GluN1C1Δ.

Primary striatal cell cultures, immunoblots, immunocytochemistry, cyclic AMP (cAMP) production assay and live calcium imaging were conducted as previously described.^14^

### Data analysis

Results are expressed as fold of control (mean±s.e.m.). ‘*N*' represents the number of independent cell culture experiments, and ‘*n*' the number of wells for a given culture or the number of mice per group. Statistical analysis was carried out using Prism 5.0 (GraphPad, University of Southampton, Southampton, UK). *T*-test, one or two-way analysis of variance, with *post hoc* analysis performed where appropriate and significance was set to *P*<0.05.

## Results

### Co-stimulation of D1R and NMDAR induces D1R/GluN1 complexes

**Figure 1:**
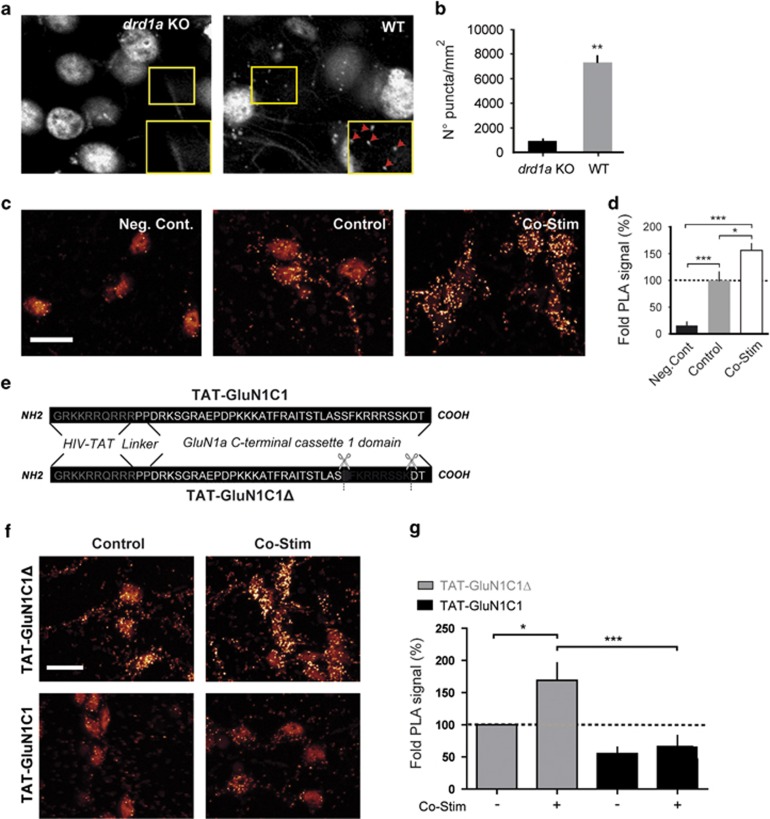
Endogenous D1R/GluN1 complexes in medium spiny neurons (MSNs) are regulated by receptor co-stimulation and blocked by TAT-GluN1C1. (**a**) Confocal images and (**b**) quantification of D1R/GluN1 complexes detected by proximity ligation assay (PLA) in the nucleus accumben (NAc) of D1R-deficient (*drd1a* KO) or wild-type (WT) mice. **P<0.01; unpaired Student's *t*-test, *n*=4. (**c**) Images and (**d**) quantification of proximity ligation assay (PLA) signals from cultured MSN obtained when the dopamine D1 receptor (D1R) antibody is omitted as a negative control (Neg. Cont), or when the D1R and GluN1 antibodies are used after a 10-min incubation in the absence (Control) or presence of 0.3 μM glutamate and 3 μM SKF38393 (Co-stim). One-way analysis of variance (ANOVA), Newman–Keuls *post hoc* test, *P<0.05; ***P<0.001; *N*=4–5 independent experiments. (**e**) Amino-acid sequence of TAT-GluN1C1 and TAT-GluN1C1Δ peptides. (**f**) PLA images and (**g**) quantifications of D1R/GluN1 complexes from MSN pre-treated with TAT-GluN1C1Δ or TAT-GluN1C1 and co-stimulated or not one-way ANOVA, Newman–Keuls *post hoc* test, *P<0.05; ***P<0.001; *n*=5–6; unpaired Student's *t*-test. Scale bar, 10 μM.

We sought to detect endogenous D1R/GluN1 complexes in their native environment in MSN by the PLA approach. A clear punctate signal was detectable in slices from naïve wild-type mice but not in *drd1a* knockout mice (*drd1a* KO; Figures 1a and b). In agreement with a strong somatic expression of both D1R and GluN1 in cultured MSN (Supplementary Figure S1a), D1R/GluN1 complexes were concentrated on the soma. The PLA signal was also specific *in vitro,* as it disappeared if one of the two primary antibodies was omitted (Figures 1c and d; Neg. Cont). To study whether D1R/GluN1 complexes are regulated by glutamate and DA inputs, cultured MSN were stimulated with glutamate (0.3 μM) or SKF38393 (3 μM) used separately (Supplementary Figure S1b and c), or together (Figures 1c and d and Supplementary Figure S1b and c), a treatment hereafter referred to as co-stim. We previously validated this co-stim model as instrumental to identify signalling events required for cocaine-induced responses *in vivo.*^14^ MSN co-stimulated for 10 min presented significantly more D1/GluN1 complexes than controls (156±12.99%, Figures 1c and d). In light of previous studies showing that agonism at the D1R can decrease^18^ or spare^17^ D1R/GluN1 interaction depending on the model used, our data further suggest that D1R/GluN1 interaction is a fine-tuned dynamic mechanism that depends, at least in part, on agonist binding and raise questions as to the functional relevance of these complexes.

To interfere with D1R/GluN1 proximity, we designed a TAT-coupled peptide corresponding to the C1 cassette (D_864_–T_900_) of GluN1 that binds to D1R (TAT-GluN1C1, Figure 1e). This peptide was cell-permeable and not deleterious for neuronal survival (Supplementary Figure S2a and b). As a control peptide (TAT-GluN1C1Δ), we eliminated nine amino acids (_890_SFKRRRSSK_898_) that are involved in the electrostatic interaction between D1R and GluN1 fragments *in vitro.*^23^ This strategy proved efficient and selective as TAT-GluN1C1 blocked the increase of D1R/GluN1 complexes induced by the co-stim (169±27%, Figures 1f and g), whereas it did not significantly change D1R/GluN1 proximity under basal conditions. The TAT-GluN1C1Δ did not prevent the increase of D1R/GluN1 complexes induced by the co-stim (Figures 1f and g). This increase of D1R/GluN1 induced by the co-stim in MSN pre-treated with the control TAT-GluN1C1Δ peptide and the inhibitory effect of the TAT-GluN1C1 were confirmed by immunoprecipitation and occurred independently on changes of expression levels of D1R or GluN1 (Supplementary Figure S1d).

### D1R/GluN1 complexes control D1R-mediated potentiation of NMDAR signalling to ERK

**Figure 2:**
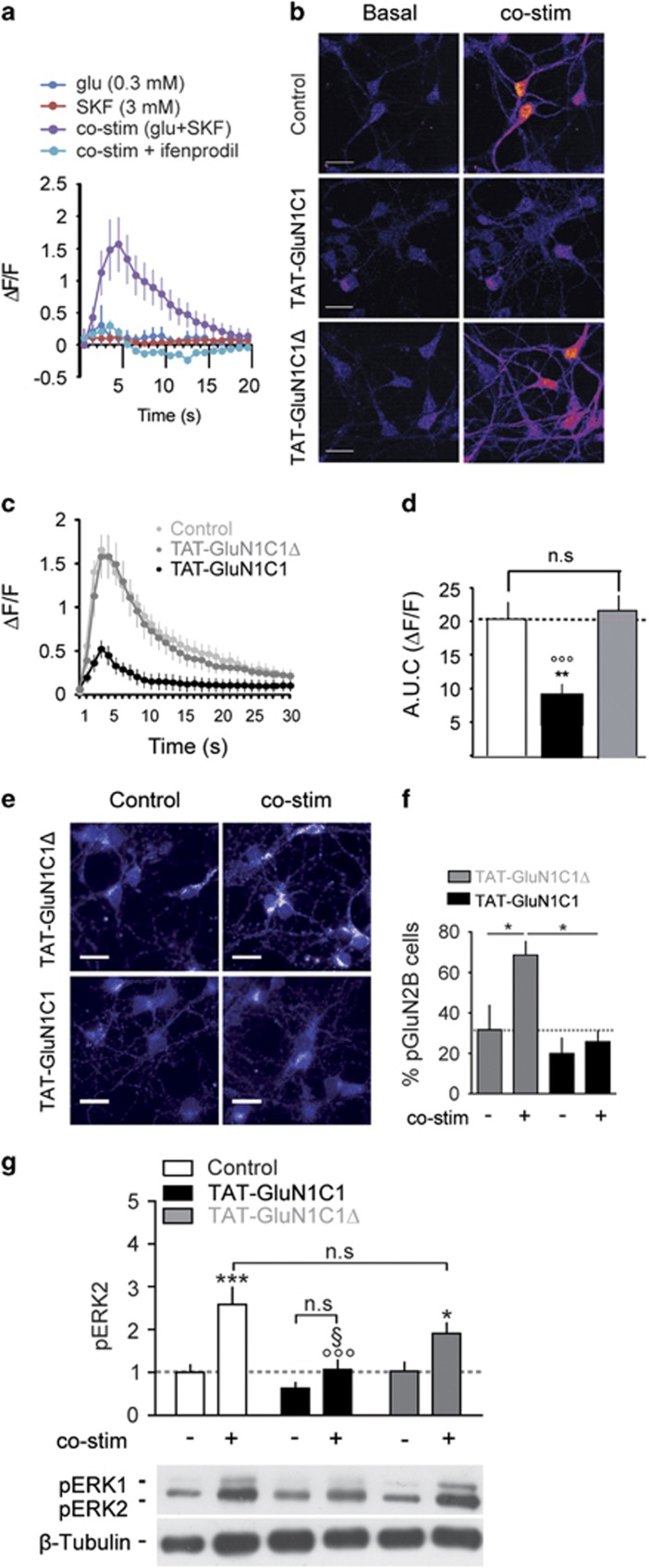
Dopamine D1 receptor (D1R)/GluN1 complexes control D1R-mediated potentiation of NMDAR signalling to extracellular signal-regulated kinase (ERK). (**a**) Calcium profiles (ΔF/F±s.e.m.) from medium spiny neurons (MSNs) treated with glutamate or SKF38393 or both (co-stim) in the absence or presence of ifenprodil (10 μM). (**b**) Images of calcium signals under basal conditions (left) and at the peak response induced by the co-stimulation in the absence (Control, top row), or presence of TAT-GluN1C1 (middle) or TAT-GluN1C1Δ (bottom). (**c**) Calcium profiles and (**d**) corresponding area under the curves (AUC) from neurons treated co-stimulated in the absence or presence of TAT-GluN1C1 (black) or TAT-GluN1C1Δ (grey); *N*=3–5, *n*=72–176 cells). One-way analysis of variance (ANOVA), Bonferroni *post hoc* test, **P<0.01; (Control); °°°*P*<0.001; (TAT-GluN1C1Δ); (**e**) Immunocytochemistry of phospho-Tyr1472-GluN2B (pGluN2B) and (**f**) percentage of pGluN2B-positive neurons from MSNs pre-treated with TAT-GluN1C1Δ or TAT-GluN1C1 and co-stimulated or not (Control) for 10 min. *P<0.05; *N*=4; one-way ANOVA, Newman–Keuls *post hoc* test. (**g**) ERK1/2 phosphorylation (pERK) measured by immunoblot from MSN co-stimulated or not in absence (white) or presence of TAT-GluN1C1 (black) or TAT-GluN1C1Δ (grey). *P<0.05; ***P<0.001 (Control); °°°*P*<0.001 (co-stim); ^§^*P*<0.05 (TAT-GluN1C1Δ, co-stim); *N*=5; two-way ANOVA, Bonferroni *post hoc* test.

We hypothesised that D1R/GluN1 interaction may underlie the convergence of DA and glutamate signals onto ERK and control striatal-dependent plasticity and behaviour. We thus evaluated the impact of TAT-GluN1C1 on the D1R-induced potentiation of NMDAR functions that trigger ERK activation in cultured MSNs. As previously described,^14^ the combined application of individually inefficient doses of glutamate and SKF38393 (co-stim) triggers transient calcium entries that we identified here as dependent on ifenprodil-sensitive GluN2B-NMDAR (Figure 2a). Interestingly, this facilitation of NMDAR functions was blunted by TAT-GluN1C1, whereas the control TAT-GluN1C1Δ peptide had no effect on amplitude and kinetics of calcium signals (Figures 2b–d).

As we previously demonstrated that this potentiation of NMDAR by D1R also involves a cAMP-independent phosphorylation of Tyr1472-GluN2B (pGluN2B),^14^ we further evaluated the role of the D1R/GluN1 complexes formation in D1R-induced phosphorylation of Tyr1472-GluN2B. Importantly, the increase of pGluN2B-positive cells induced by the co-stim in the presence of the control TAT-GluN1C1Δ was abrogated by TAT-GluN1C1 (Figures 2e and f). Downstream from calcium, TAT-GluN1C1 selectively blocked ERK activation induced by the receptor co-stim (Figure 2g) in a dose-dependent manner (Supplementary Figure S2c), thus supporting that D1R/GluN1 complexes act a molecular bridge linking DA to glutamate signalling in MSN.

**Figure 3:**
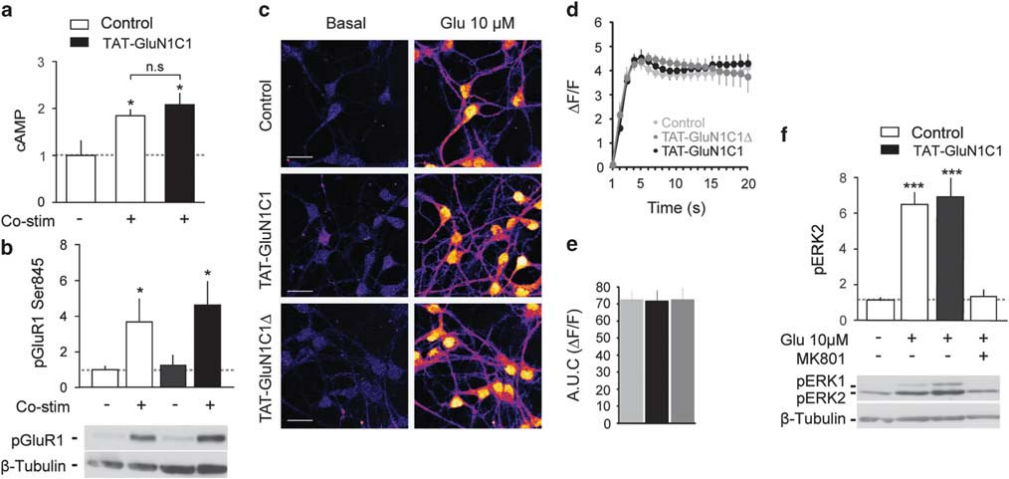
Canonical individual dopamine D1 receptor (D1R) or NMDAR signalling is preserved in presence of TAT-GluN1C1. (**a**) Fold increase in cyclic AMP (cAMP) production 10 min after the co-stimulation in the absence (white) or presence of TAT-GluN1C1 (black); *N*=2; *n*=3; one-way analysis of variance (ANOVA), Newman–Keuls *post hoc* test. (**b**) Immunoblots and quantification of Ser^845^-GluA1 phosphorylation (pGluA1) from medium spiny neurons (MSNs) co-stimulated or not in the absence (white) or presence of TAT-GluN1C1 (black); *N*=5; two-way ANOVA, Bonferroni *post hoc* test. (**c**–**e**). As for Figures 2b–d, but MSNs are stimulated with glutamate (10 μM). (**f**) Phosphorylated extracellular signal-regulated kinase (pERK) immunoblots and quantification from cells stimulated or not with 10 μM glutamate (10 min) in the absence or presence of TAT-GluN1C1 or MK801. *N*=7–8; one-way ANOVA, Bonferroni *post hoc* test. (**a**–**f**) *P<0.05; ***P<0.001 (control).

Since the TAT-GluN1C1 has never been used to alter endogenous D1R/GluN1 complexes (see discussion), we had to exclude the possibility that it may non-specifically interfere with D1R and/or NMDAR signalling independently of their heteromerization. TAT-GluNC1 binds to the t2 domain of D1R (Supplementary Figure S3a, d), which does not overlap with the third intracellular loop required for the positive coupling of D1R to adenylyl cyclase activation^24^ and cAMP production through the Gs protein Golf.^25^ As expected, cAMP production induced by the co-stim was unaffected by TAT-GluN1C1 (Figure 3a). Moreover, TAT-GluN1C1 preserved the downstream increase in Ser^845^ phosphorylation of the GluA1 subunit of AMPAR (Figure 3b), a well-established target of the D1R/cAMP/protein kinase A (PKA) cascade.^26^ We also ruled out potential side effects on NMDAR signalling by showing that glutamate-induced calcium increase and downstream NMDAR-dependent ERK activation were unaffected by TAT-GluN1C1 (Figures 3c–f). Disruption of D1R/GluN1 complexes thus blocks the crosstalk linking D1R to the potentiation of calcium influx through GluN2B-NMDAR and downstream ERK activation, an important event for cocaine-mediated responses *in vivo*.

### D1R/GluN1 complexes govern long-term striatal plasticity

**Figure 4:**
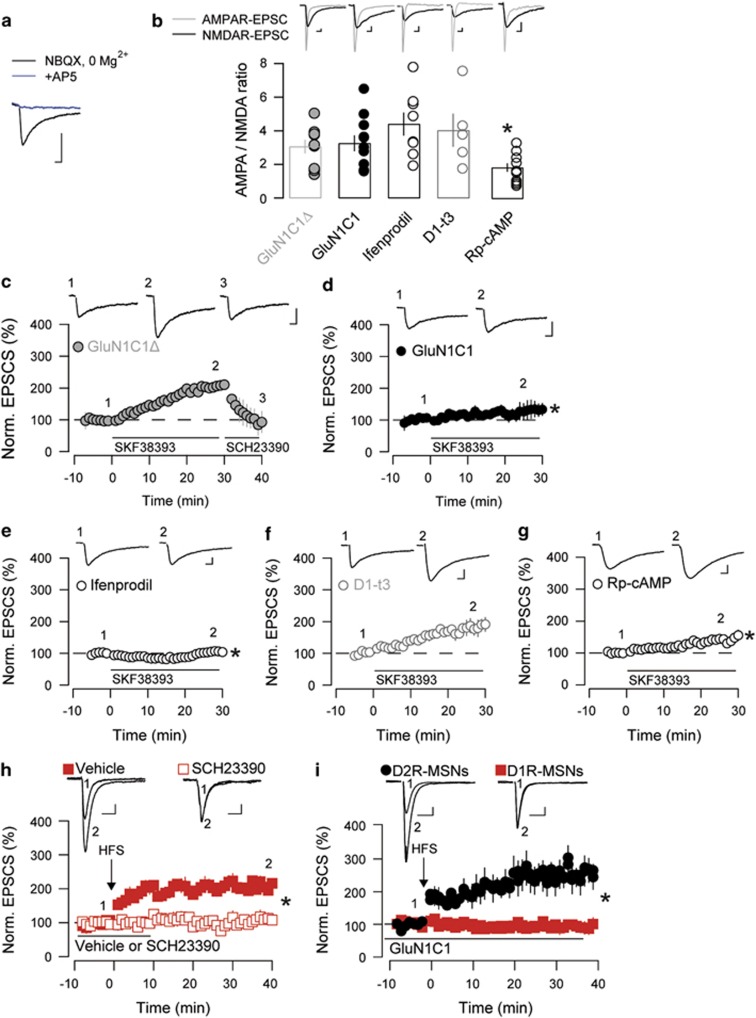
Dopamine D1 receptor (D1R)/GluN1 complexes control long-term synaptic plasticity in D1R-medium spiny neurons (MSNs). (**a**) NMDAR-mediated excitatory post synaptic currents (EPSCs) were pharmacologically isolated (blockade of AMPAR or NMDAR-EPSC by NBQX and by AP5, respectively). (**b**) Example traces and quantification of AMPA/NMDAR ratio after AMPAR- and NMDAR-EPSCs were recorded successively (before and after pharmacological isolation of the NMDAR-EPSC) in presence of GluN1C1, or GluN1C1Δ, D1-t3, RpcAMP in the patch pipette or bath application of the GluN2B-containing NMDAR antagonist, ifendprodil (*n*=6–10). Students unpaired *t*-test, *P<0.05 different from control (GluN1C1Δ). (**c**) The potentiation of NMDAR-mediated EPSCs by SKF38393 observed in the presence of GluN1C1Δ in the internal solution is reversed by bath application of a D1R antagonist (SCH-23390 10 μM; *n*=9). (**d**) In the presence of GluN1C1, SKF38393 no longer potentiates NMDAR-EPSCs (*n*=10). (**e**) Ifenprodil prevents the SKF38393-induced potentiation of NMDAR-mediated EPSCs (*n*=8), whereas the D1-t3 peptide (*n*=5) has no effect (**f**). (**g**) RpcAMP partially, but significantly, alters the potentiation NMDAR-mediated EPSCs by a D1R agonist (*n*=12). (**h**) The D1R antagonist (SCH-23390 10 μM; *n*=6) prevents high-frequency stimulation-induced (HFS, 100 Hz) long-term potentiation (LTP) of AMPAR-EPSCs recorded in D1R-MSNs (*n*=6). (**i**) GluN1C1 prevents HFS-LTP in D1R-MSNs (*n*=12) but not in D2R-MSNs (*n*=6). (**a**–**i**) MSNs were identified using *drd1a*-eGFP, *drd1a-td*Tomato or *drd2*-eGFP transgenic mice. *P<0.05; Students unpaired *t*-test when percent variation from baseline during the last 5 min is different from control condition. Scale bars, 10 ms, 20 pA.

Given the importance of long-term plasticity at glutamatergic synapses onto MSN for behavioural responses to cocaine, we studied whether D1R/GluN1 complexes were involved in the modulation of glutamatergic transmission in the NAc in slices prepared from transgenic mice expressing tdTomato or the eGFP under the control of the promoter of the *drd1a* gene to identify D1R-MSN.^22^ D1R/GluN1 proximity did not seem primarily involved in basal glutamatergic synaptic transmission since neither peptide modified the AMPA/NMDA ratio (Figures 4a and b). We then tested if D1R/GluN1 complexes are involved in the facilitation of NMDAR-mediated EPSCs by D1R agonists.^27^ This appeared to be the case since the D1R agonist-induced increase in NMDAR-EPSCs amplitude in the presence of GluN1C1Δ in the patch pipette (Figure 4c) was fully blocked with GluN1C1 (Figure 4d). Similarly, to the potentiation of NMDAR-dependent calcium influx evoked by the D1R stimulation in cultured MSN (Figure 2a), ifenprodil blunted the D1R agonist-induced potentiation of NMDA-EPSCs, thus supporting that D1R/GluN1 may preferentially modulate GluN2B-containing NMDAR (Figure 4e). In agreement with this, a peptide sequence corresponding to the binding domain of D1R to GluN2A (D1-t3)^18^ had no effect on the facilitation of NMDA-EPSCs induced by D1R stimulation (Figure 4f). To further characterize the mechanism underlying the facilitation of NMDA currents by D1R in our model, we also assessed the role of PKA activity downstream from D1R. We found that the PKA inhibitor, RpcAMP, strongly altered basal glutamatergic transmission as seen with the marked reduction of the AMPA/NMDA ratio (Figure 4b) and partially, but significantly, inhibited the D1R agonist-induced potentiation of NMDA-EPSCs (Figure 4g). However, this PKA-dependent component of the D1R-mediated NMDAR functions is unlikely to be involved in the triggering of ERK activity downstream of both receptors, which is a cAMP-independent event.^14^

With regard to long-term synaptic plasticity, high-frequency stimulation-LTP induced in identified D1R-MSN strictly depends on both NMDAR and ERK activation.^15^ Here we found that it was abrogated by a bath application of the D1 antagonist SCH-23390 (Figure 4h). In slices from *drd1a*-eGFP and *drd2*-eGFP mice, high-frequency stimulation-induced LTP in both D1R- and D2R-MSN.^15^ Importantly, addition of GluN1C1 in the patch pipette selectively blunted LTP in D1R-MSN, but not in D2R-MSN (Figure 4i). This results thus show that D1R/GluN1 interaction controls a ERK-dependent synaptic plasticity in D1R-MSN specifically.

### D1R/GluN1 complexes in the NAc control cocaine-induced adaptation *in vivo*

**Figure 5:**
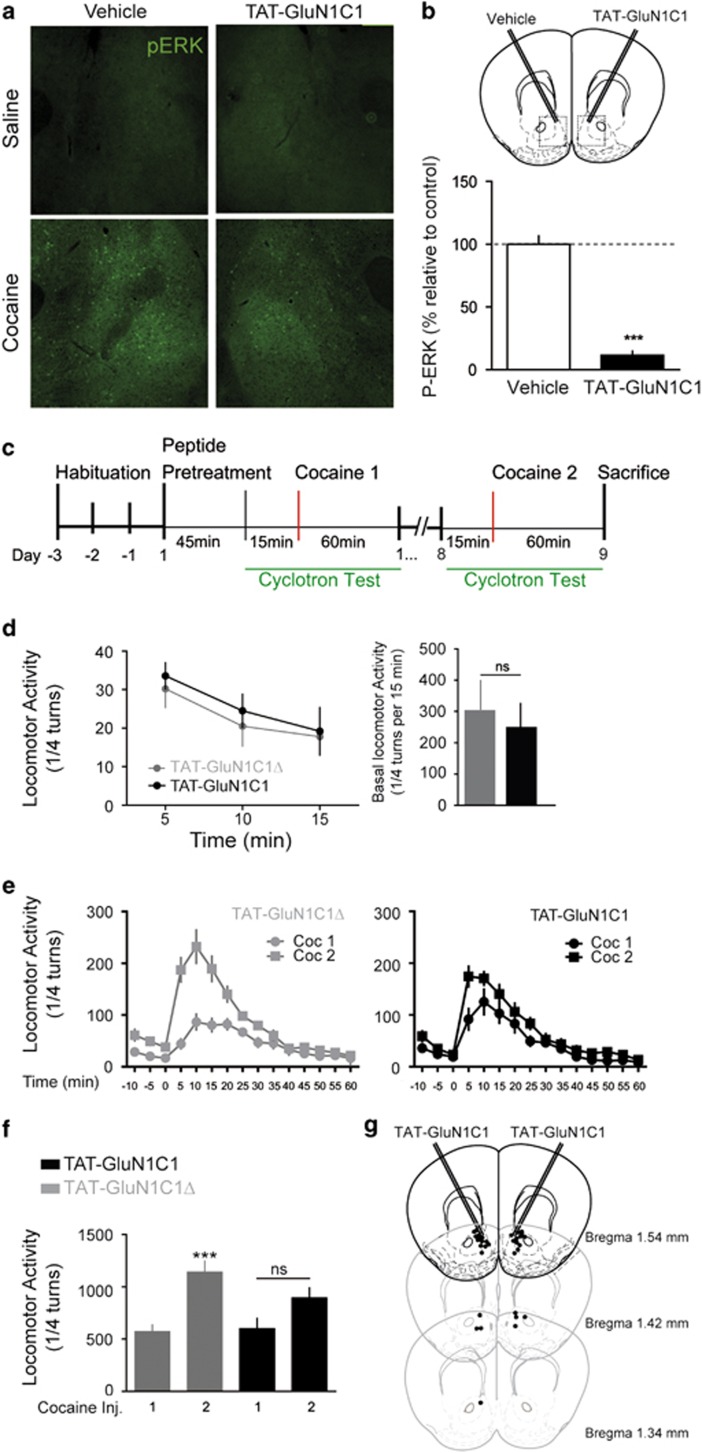
TAT-GluN1C1 infusions into the nucleus accumbens (NAc) alter cocaine-induced adaptations *in vivo*. (**a**) Immunohistochemical detection of phosphorylated extracellular signal-regulated kinase (pERK) in the NAc shell of mice that received unilateral TAT-GluN1C1 infusion 1h before saline or cocaine and were killed 10 min later. (**b**) Percentage of pERK-positive striatal cells normalised to the vehicle-treated NAc. ***P<0.001, Students paired *t*-test (*n*=4). (**c**) Diagram of the locomotor sensitization protocol. (**d**) Time course (left) and total (right) basal locomotor activity before the first cocaine injection in the TAT-GluN1C1Δ (grey, *n*=9) and TAT-GluN1C1-treated groups (black, *n*=8). (**e**) Acute cocaine injection (15 mg kg^−1^) induced a similar increase in locomotor activity in both groups (Coc 1). A second injection (Coc 2) induced a significant sensitization of the locomotor response only in the control group (TAT-GluN1C1Δ, left) but not in mice pre-treated with TAT-GluN1C1 (right). ***P<0.001 (Coc 1); one-way analysis of variance (ANOVA), Bonferroni *post hoc* test. (**d**–**f**) n.s., not significant. (**g**) NAc injection sites according to the atlas of Paxinos and Franklin (2001).

To examine whether D1R/GluN1 complexes have a functional relevance *in vivo*, we infused unilaterally TAT-GluN1C1 into the NAc of mice and found it significantly lowered the number of pERK-positive cells induced by cocaine when compared with the contra-lateral NAc (Figures 5a and b).

As a causal connection between cocaine-evoked ERK activation, synaptic potentiation and behaviour has been established for locomotor sensitization,^12,15^ we investigated the impact of a bilateral infusion of TAT-GluN1C1 or TAT-GluN1C1Δ into the NAc using the two-injection protocol of sensitization^13^ (Figure 5c). In both groups of mice, basal locomotor activity (Figure 5d) and acute increase in activity induced by a single cocaine injection (Figures 5e and f; cocaine Inj.1) were not statistically different. Mice received a second injection of cocaine at day 8 and the TAT-GluN1C1Δ pre-treated mice showed a clear behavioural sensitization. In contrast, the TAT-GluN1C1-pre-treated group did not show any significant increase in their locomotor activity when comparing day 1 and day 8 (Figures 5f and g). Therefore the dissociation of D1R/GluN1 interactions remarkably prevents the development of psychomotor sensitization to cocaine but not basal locomotion or acute responses to cocaine.

## Discussion

This work provides evidence on multiple levels that dynamic associations between D1R and GluN1 are involved in signalling, plasticity and behaviour that model early phases of cocaine addiction. A selective inhibition of D1R/GluN1 complexes is possible without compromising the functions of individual D1R and NMDAR, which possibly avoids the caveat of frequent side effects encountered due to global blockade of either given receptor subtype.

The strategy we used to block D1R/GluN1 proximity fundamentally also tells us more about the mechanisms involved in D1R/GluN1 interaction, as it confirmed a physiological relevance of the Arg-rich epitope in C1, as predicted by Woods *et al.*^23^ *in vitro*, but here in the receptor's native environment. This epitope shares remarkable similarities with the Arg-rich epitope involved in physical interactions of D2R with Adenosine A2 receptors,^28^ which suggests that conserved electrostatic interactions may be involved. We chose to use the full-length C1 cassette sequence, the TAT-GluN1C1 peptide, to gain more specificity in targeting D1R/GluN1 complexes and to compare our data with previous studies. However, we did test a peptide containing only the Arg-rich epitope (TAT-GluN1C1-min) and found it was sufficient to block ERK activation by receptor co-stim (Supplementary Figure S3a, c, f). Importantly, previous studies have used the complementary interaction site to design interfering peptides.^20^ This site corresponds to the sequence of the D1RcT that binds to GluN1 (TAT-D1-t2). When we tested the TAT-Dt-2, it tended to increase basal ERK activity (*N*=4; *P*=0.055; Supplementary Figure S3a, d, g). This surprising impact on NMDAR-dependent signalling to ERK may result from a competition between TAT-D1-t2 and GluN1 for binding to Calmodulin^18,29^ but requires further investigation. Likewise, the peptide TAT-D1-t3, which prevents D1R binding to the GluN2A subunit, did not block ERK activation by co-stim of NMDAR and D1R (Supplementary Figure S3a, e, h), which is agreement with its lack of effect on D1R-induced facilitation of NMDA-EPSCs (Figure 4f). While other binding partners of the C-terminal domains of D1R and NMDAR probably also contribute to the processes we examine herein, no other known partner could reproduce the observed effects here on the multiple levels and different readouts. Such partners include the aforementioned calmodulin; however, interference with this interaction would likely prolong or enhance NMDAR signalling, opposite to what we observe for TAT-GluN1C1, and this site has a low affinity in comparison with the binding domain in C1.^29^ Other known partners of the C1 domain of the GluN1 subunit include scaffolding proteins, such as yaotiao, neurofilaments and microtubules.^30^ As our electrophysiology results showed no change in NMDAR current at basal levels (Figure 4), there is ample reason to doubt that TAT-GluN1C1 is sufficient to perturb the trafficking of NMDAR guided by these scaffold protein interactions. As for the D1R C-terminal partners, TAT-GluN1C1 could possibly compete with PSD-95 for its binding to D1R; however, we did not see any change in cAMP production (Figures 3a and b), which suggests that D1R remains expressed at the surface and capable of signalling. The region of interaction with PSD-95 may well rather depend on the D1-t1 or D1-t3 regions (and not D1-t2 which is examined here). The t2 domain of D1R also interacts with N-ethylmaleimide-sensitive factor.^31^ N-ethylmaleimide-sensitive factor is a hexametric ATPase studied for its functions in membrane trafficking and endocytosis. Interfering with this interaction blocked D1R membrane localisation and inhibited D1R-mediated cAMP production, which was not observed in the present study. Altogether, our data support the idea that the strategy we chose to disrupt D1R/GluN1 complexes with TAT-GluN1 does not drastically alter the coupling of D1R and NMDAR to their cognate signalling partners, but specifically impact the interplay between D1R and NMDAR-dependent signalling.

The D1R/NMDAR interplay is critical for striatal signalling and plasticity.^32^ An important mechanism for this dialogue between both receptors involved a D1R-mediated phosphorylation of GluN2B that is important for cocaine-induced signalling and behaviour.^14^ The fact that TAT-GluN1C1 blocked (1) the phosphorylation of GluN2B, (2) the potentiation of NMDAR-EPSCs; (3) the potentiation of NMDAR–calcium influx by D1R and (4) ERK activation downstream of both receptors supports the idea that D1R/GluN1 interaction has a gating function for the facilitation of NMDAR-dependent calcium influx triggering ERK activation in response to D1R stimulation.

The observation that TAT-GluN1C1 spared the canonical cAMP/PKA pathway downstream from D1R is in agreement with previous work in cell lines overexpressing D1R and GluN1 where disruption of their interaction did not modify D1R-induced cAMP production *per se,* but rather its potentiation by NMDA,^19^ a phenomenon that we did not observe in our system (data not shown). It is also in favour of an uncoupling between cAMP/PKA and ERK activation in MSN that could account for the preserved ERK phosphorylation by psychostimulants seen previously in mice deficient for the G_α_olf/cAMP/PKA pathway^33^ (Gnal+/−). We thus propose that a trigger for ERK activation relies on D1R/GluN1 complexes that recruit D1R-mediated signalling onto GluN2B and promotes calcium influx. The duration and amplitude of ERK activation would then be modulated by the D1R/cAMP/PKA cascade *via* DARPP-32 and inhibition of phosphatases targeting the ERK pathway.^34^

A causal link between ERK activation, long-term synaptic plasticity in D1R-MSNs and behavioural responses to cocaine has recently been established. A single cocaine injection indeed triggers a potentiation of AMPAR-mediated EPSCs in D1R-MSN and optogenetically induced depotentiation of these excitatory inputs onto MSN abolishes locomotor sensitization to cocaine.^15^ Herein, we established that D1R/GluN1 complexes control the integration of DA and glutamate inputs by MSN, long-term synaptic plasticity and locomotor sensitization to cocaine. Genetic or pharmacological inhibition of GluN2B and GluN2A has been shown to differentially alter the potentiation of NMDAR by a D1R agonist in MSN. Whereas GluN2A knockout mice showed a higher potentiation of NMDAR-EPSCs by D1R, pharmacological inhibition of GluN2B drastically inhibited this facilitation.^35^ The demonstration here that D1R/GluN1 complexes are necessary both for the phosphorylation of GluN2B (Figures 2e and f) and the facilitation of ifenprodil-sensitive NMDAR-EPSCs (Figures 4d and e), strongly argues in favour of a privileged role of these complexes in the D1R-mediated potentiation of GluN2B-NMDAR in MSN.

Although further studies will be required to determine whether the synergistic effect of D1R/NMDAR interaction on signalling results from crosstalk or oligomerization,^36^ a growing body of evidences suggests that oligomerization can affect activity or signalling but also the binding properties of receptors.^37^ A better understanding of the nature of D1R/NMDAR interaction could open new routes toward the development of more specific ligands that could selectively target D1R/NMDAR complexes. Our results highlight the potential for future research to target D1R/NMDAR complexes as a valuable therapeutic approach in drug addiction.

By contrast to the mechanism of D1R-mediated potentiation of NMDAR functions through D1R/GluN1 oligomers we describe here, cocaine was previously shown to induce D2R/GluN2B oligomer formation, which reduced NMDAR-mediated currents.^38^ In the striatum, the association between the third intracellular loop of the D2R and the carboxyl terminal tail of GluN2B was enhanced by cocaine. The binding of D2R to the GluN2B displaces CaMKII and reduces GluN2B phosphorylation at Ser^1303^ as well as calcium currents. Furthermore, this interaction was shown to have functional consequences as a TAT-coupled peptide that mimicked the D2R region of interaction prevented acute horizontal activity and stereotyped behaviour normally induced by high doses of cocaine. However, the role of D2R/GluN2B complexes in the development of behavioural sensitization to cocaine is not yet defined. The fine tuning of DA and glutamate crosstalk achieved by receptor complexes should thus be a novel consideration for understanding the pathophysiology of the vast spectrum of psychiatric disorders associated with improper dopaminergic modulation of glutamate transmission in brain regions where the existence of D1R/GluN1 complexes is strongly supported.^39^
