# Supplementary material for: D1R/GluN1 complexes in the striatum integrate dopamine and glutamate signalling to control synaptic plasticity and cocaine-induced responses
Source: Mol Psychiatry. 2014 Jul 29;19(12):1295–304. doi: 10.1038/mp.2014.73 (PMC4255088; doi:10.1038/mp.2014.73)
Supplement: Supplementary Information [file mp201473x1.doc]

**SUPPLEMENTARY MATERIALS AND METHODS**

**Chemicals and reagents**

The following pharmacological agents were added to cultured MSN 30min prior to and during treatments: the GluN2B antagonist ifenprodil (α-(4-hydroxyphenyl)-β-methyl-4-benzyl-1-piperidineethanol (+)-tartrate salt, 10μM) from Sigma Aldrich (St. Louis, MO); NMDA receptor antagonist, (+)-MK801 ((5R,10S)-(+)-5-methyl-10,11-dihydro-5H-dibenzo[a,d]cyclohepten-5,10-imine hydrogen maleate, 10μM) from Tocris Bioscience (Ellisville, MO).

For electrophysiology, brain slices were superfused, when indicated, with 10µM of the D1R antagonist, R(+)-SCH23390(R(+)-7-chloro-8-hydroxy-3-methyl-1-phenyl-2,3,4,5-tetrahydro-1H-3-benzazepine hydrochloride), 20µM of the AMPAR antagonist NBQX (2,3-dihydroxy-6-nitro-7-sulfamoyl-benzo[f]quinoxaline-2,3-dione) or 50µM of DL-AP5 (DL-2-Amino-5-phosphonopentanoic acid) or ifenprodil (α-(4-hydroxyphenyl)-β-methyl-4-benzyl-1-piperidineethanol (+)-tartrate salt, 10μM) from Sigma Aldrich, diluted in cooled artificial cerebrospinal fluid (ACSF) containing (in mM): 119 NaCl, 2.5 KCl, 1.3 MgCl2, 2.5 CaCl2, 1.0 Na2HPO4, 26.2 NaHCO3 and 11 glucose, bubbled with 95% O_2_ and 5% CO_2_. The GluN1C1, GluN1C1∆ and D1-t3 peptides where diluted at 5µM in the internal solution of the patch-pipette 15min prior to and during recordings. The Rp cAMP (Rp-Cyclic 3′,5′-hydrogen phosphorothioate adenosine triethylammonium salt, Tocris) was used in the same conditions at 10µM.

**Image acquisition and quantification**

For PLA experiments, image acquisition was carried out using 63X-oil immersion objective on a SPE confocal microscope (Leica, Wetzlar, Germany). Images were acquired using a z-stack of 0.5µm intervals. Conditions were run in duplicate and quantifications were made from at least 6 images per condition; representing at least two independent experiments. Maximum projection images were analyzed using ImageJ (National Institutes of Health, Bethesda, MD) to quantify PLA punctate signal. Images were smoothed and a threshold to distinguish signal from background fluorescence was applied equally to all images and the number of puncta quantified using the “Analyze Particles” macro with the exclusion criteria of size of objects being greater than 5µm^2^.

Pictures of phospho-ERK1/2 immunohistochemical staining (magnification, 20X) were taken with a Leica DM LB fluorescence microscope coupled to a CCD. Images covering the entire surface of the NAc core and shell were acquired on both sides of the brain. The assembly of all pictures to reconstruct the entire NAc was performed with AdobePhotoshop® software. The number of phospho-ERK1/2 immunopositive cells detected at an intensity threshold defined above basal pERK levels, and kept constant for each slice, was determined automatically using the ImageJ analyze particles macro within the NAc delineated as the region of interest. The number of pERK highly-positive cells counted in the striatum infused with TAT-GluN1C1 was normalized to the number of cells counted in the corresponding contralateral striatum.

**Co-immunoprecipitation**

Experiments were performed as previously described^18^. Briefly, cultured neurons were homogenized in 200 μl of lysis buffer: 50 mM Tris-HCl (pH 7.6), 150 mM NaCl, 1% IgepalCa630, 0.5% sodium deoxycholate, 2 mM EDTA, 1 mM sodium orthovanadate, 1 mM PMSF and a cocktail of protease inhibitors (Roche diagnostics GmbH, Mannheim, Germany) and centrifuged at 10,000g, 4°C, for 20 min. The supernatant was then supplemented with 1% triton X-100. A volume of extract corresponding to 700 μg of protein was incubated in the presence or absence of 2 μg of GluN1 antibody (Millipore, Billerica, Massachusetts, USA) for 4h under gentle agitation at 4°C. Protein A/G PLUS-Agarose (40 μl per co-IP; Santa Cruz, Santa Cruz, CA) was then added overnight. Pellets were washed four times for 10 minutes, boiled for 90 sec and subjected to the immunoblotting procedure using primary antibodies directed against GluN1 (BD Pharmingen, San José, CA, USA) and D1R (Sigma Aldrich)

**SUPPLEMENTARY FIGURE LEGENDS**

**Figure S1.** D1R and GluN1 co-localise and form complexes in cultured MSN that are regulated by agonists. (**a**) Co-staining of D1R (left panel) and GluN1 (center) revealed a strong proximity and co-localisation (right) in MSN. (b) Representative images of the detection of D1R/GluN1 complexes by PLA from cultured neurons treated or not with glutamate 0.3 µM or SKF38393 3 µM or both (co-stim) for 10 min. Staining was practically absent if one antibody of the pair was omitted from the PLA protocol as a negative control (Neg. Cont.). (**c**) Quantification of PLA signal after stimulation with agonists. n = 4-5; One-way ANOVA, Newman Keuls post-hoc test; *p < 0.05, **p < 0.01, versus control. (**d**) Representative GluN1 and D1R immunoblots performed from 70 µg of protein prepared from cultured MSN pre-treated with either TAT-GluN1C1Δ or TAT-GluN1C1 and co-stimulated or not (left panel). The β-tubulin staining serves as a loading control. Note that expression levels of GluN1 and D1R do not significantly vary between experimental groups. The fold expression of GluN1 relative to the control group pre-treated with TAT-GluN1C1Δ (group 1) is 0.89 ± 0.106 for group 2, 1.10 ± 0.105 for group 3 and 1.06 ± 0.112 for group 4; N = 3. For D1R the fold expression relative to group 1 is 0.89 ± 0.19 for group 2, 0.96 ± 0.17 for group 3 and 0.9 ± 0.16 for group 4. Representative co-immunoprecipitation of GluN1 and D1R performed from the same 4 experimental groups of MSN described above in the presence or absence (No 1°Ab) of GluN1 antibody (right panel). Following immunoprecipitation of GluN1, samples were processed for immunoblotting with both GluN1 and D1R antibodies. Note that the co-stimulation increased the co-immunoprecipitation of GluN1 and D1R that that is prevented in the presence of TAT-GluN1C1.

**Figure S2.** TAT-GluN1C1 penetrates into MSN, is not toxic, and dose dependently blocks ERK activation induced by a co-stimulation of D1R and NMDAR. (**a**) Neurons were incubated for 1h in the absence (first row) or presence of 5 µM of the unconjugated (center) or biotin-coupled (bottom) versions of the TAT-GluN1C1 peptide. The morphology of the neurons was visualized by immunofluorescence with anti-MAP2 antibody (red) and the penetration of biotin-coupled peptide was detected with streptavidin Alexa Fluor 488 (green). The nuclei were counterstained with Hoechst (blue). Shown are representative confocal pictures of fluorescent staining together with phase contrast. The streptavidin labeling gives rise to signal specifically within neurons pre-treated with the biotin-coupled TAT-GluN1C1. (**b**) Bars are the means (± s.e.m) of % cell survival (N = 3) quantified in neurons incubated or not with TAT-GluN1C1 and measured on the basis of the morphology of the nuclei visualized by the Hoechst; No statistical significance found by unpaired Student’s t-test. (**c**) Bars are means (± s.e.m) of Thr202-Tyr204-ERK1/2 phosphorylation (pERK) normalized to un-stimulated cells. The relative increase in pERK2 induced 10min after the co-stimulation (4.09 ± 0.489, N = 7) is diminished in the presence of 1µM of TAT-GluN1C1 (3.46 ± 1.06, N = 5) and significantly blocked at the dose of 5µM (1.57 ± 0.342, N = 7). Two-way ANOVA revealed a significant effect of stimulation (F_1,32_= 31.13, *** p < 0.0001) and an interaction between peptide concentration and stimulation (F_2,32_= 4.153, *p< 0.05). Bonferroni post-test revealed a significant reduction of ERK activation induced by the co-stimulation in the 5µM treated group versus the 1 µM treated group (^§^P <0.05) and untreated group (°°°p < 0.0001).

**Figure S3:** Impact of TAT-GluN1C1min, TAT-D1-t2 and TAT-D1-t3 on ERK activity induced by a co-stimulation of D1R and NMDAR in cultured MSN. (**a**) Diagram depicting the mechanism of action and interaction domains of GluN2A, GluN1 and D1R directed peptides. (**b-e**) Schematic representation of TAT-GluN1C1, TAT-GluN1C1-min, TAT-D1-t2 and TAT-D1-t3 peptide sequences used in this study. (**f-h**) Representative immunoblots and quantification of normalized ERK phosphorylation performed on cultured MSN pre-treated or not with 5 µM of (**f)** TAT-GluN1C1min peptide 1h before an incubation of 10 min in the absence or presence of 0.3µM glutamate and 3µM SKF-38393 (co-stim); N = 4; Two-way ANOVA, Bonferroni post-hoc test; *p < 0.05 versus control, (**g**) TAT-Dt-t2; N = 5; Two-way ANOVA, Bonferroni post-hoc test; **p < 0.01, (**h**) TAT-Dt-t3, N = 7; Two-way ANOVA, Bonferroni post-hoc test; *p <0.05.
