# Supplementary figures and images for: D1R/GluN1 complexes in the striatum integrate dopamine and glutamate signalling to control synaptic plasticity and cocaine-induced responses
Source: Mol Psychiatry. 2014 Jul 29;19(12):1295–304. doi: 10.1038/mp.2014.73 (PMC4255088; doi:10.1038/mp.2014.73)

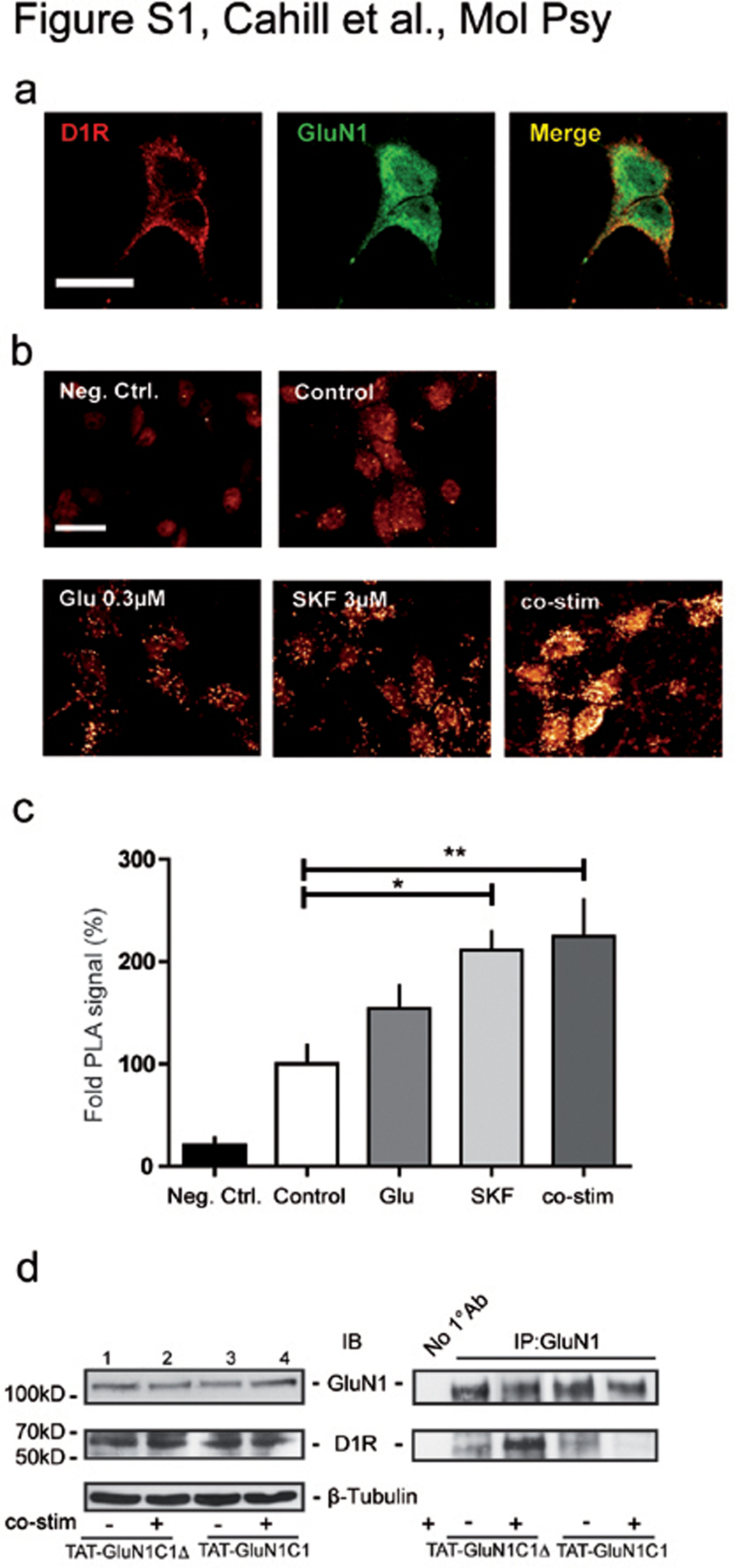

Supplement: Supplementary Figure S1 [file mp201473x2.tif]

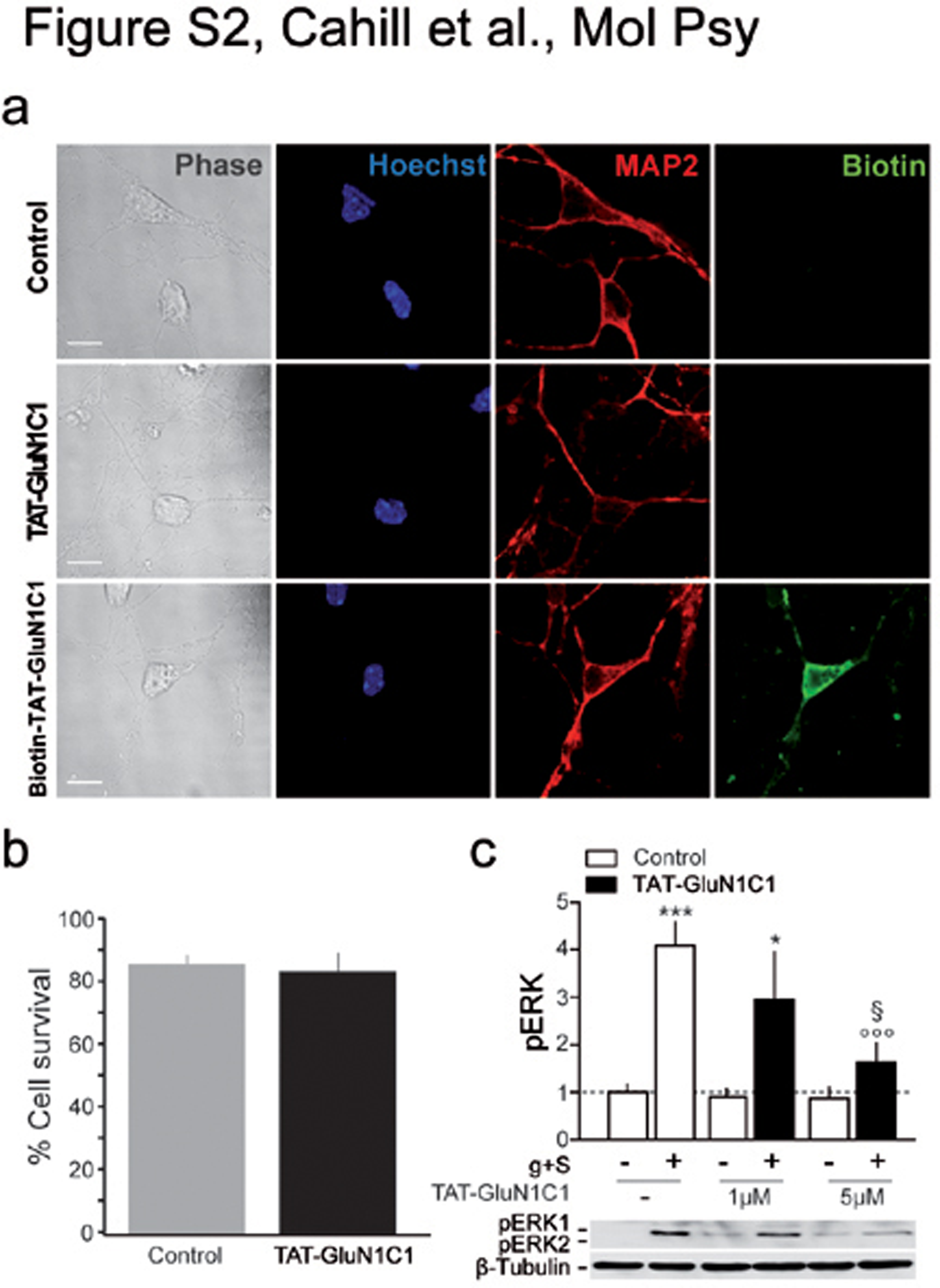

Supplement: Supplementary Figure S2 [file mp201473x3.tif]

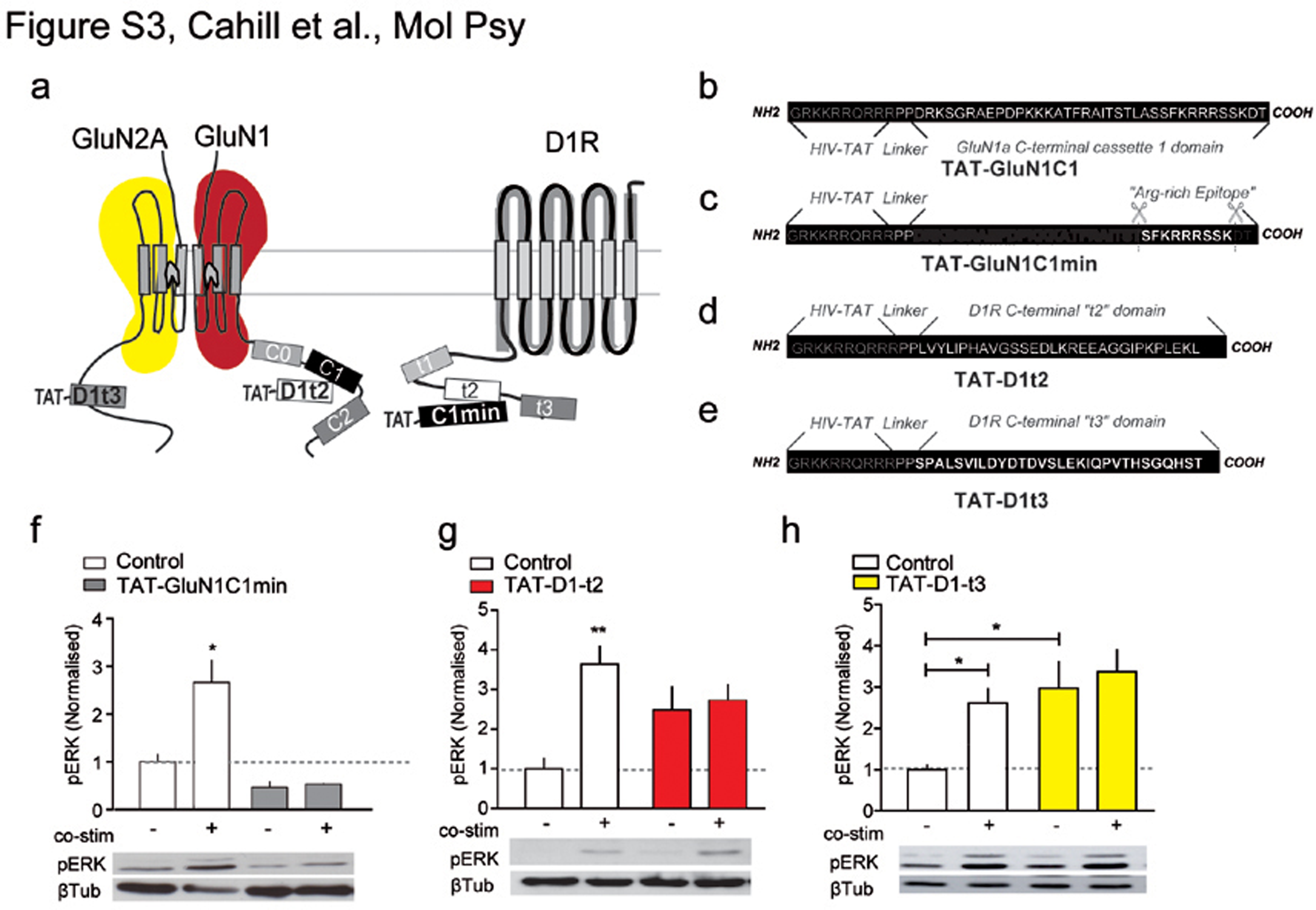

Supplement: Supplementary Figure S3 [file mp201473x4.tif]
